# Strain-Dependent Protective Innate Immunity Against *Pneumocystis* Pneumonia in Mice

**DOI:** 10.3390/jof12040239

**Published:** 2026-03-26

**Authors:** Paul C. Inzerillo, Francis Gigliotti, Terry W. Wright

**Affiliations:** Department of Pediatrics, Infectious Diseases, University of Rochester School of Medicine and Dentistry, Rochester, NY 14642, USA

**Keywords:** *Pneumocystis* pneumonia, opportunistic pathogen, innate immunity, macrophage

## Abstract

*Pneumocystis* is a respiratory fungal pathogen that causes life-threatening pneumonia in immunocompromised patients. While *Pneumocystis* can colonize healthy hosts by resisting and transiently evading innate immunity, a functional adaptive immune response is essential to prevent progressive infection. Impairments in adaptive immunity, particularly defects in CD4^+^ T cell function, are strongly associated with the development of severe *Pneumocystis* pneumonia (PCP) in humans and a wide range of mammalian species. Immune activation by *Pneumocystis* has strong genetic determinants, and a major gap in our understanding of PCP pathogenesis lies in uncovering the mechanisms by which *Pneumocystis* escapes alveolar macrophages and evades pulmonary innate immunity. Prior research determined that FVB/NJ mice display an unusual resistance to *Pneumocystis* infection. Further susceptibility testing across several inbred mouse strains revealed that the AKR/J strain, which is phylogenetically distant from the FVB/NJ strain, also exhibits a rarely described form of protective innate immunity against PCP. Notably, the mechanism of AKR/J resistance does not require CD4^+^ or CD8^+^ T lymphocytes. However, depleting alveolar macrophages prior to infection rendered AKR/J mice susceptible to PCP, highlighting the critical role of macrophages for this protective innate immune response. These novel findings establish the AKR/J inbred strain as a valuable model for investigating the interaction between *Pneumocystis* and macrophages, offering a unique opportunity to explore how these interactions lead to differential outcomes between resistant and susceptible mouse strains. Additionally, it may offer key insights into the mechanisms by which *Pneumocystis* evades macrophage-mediated innate immunity in the majority of mammalian hosts, including humans.

## 1. Introduction

Fungal infections, including those caused by *Pneumocystis jirovecii* (*P. jirovecii*), are an underappreciated threat to public health throughout the world [1,2,3,4,5,6]. *P. jirovecii* is a respiratory fungal pathogen that causes life-threatening pneumonia in patients suffering from various conditions that impair certain aspects of adaptive immunity [7,8]. Both the general term *Pneumocystis* Pneumonia (PCP), which refers to pneumonia caused by any *Pneumocystis* species, and *P. jirovecii* Pneumonia (PJP), which refers specifically to the disease caused by the human pathogen, are found in the medical and research literature. *Pneumocystis* species cannot be cultured *ex vivo*, and, to date, the only identified reservoir of infectious *P. jirovecii* is the human lung. Human-to-human transmission is the presumed mode of exposure, and although immunocompromised patients are highly susceptible to developing active PCP, *P. jirovecii* also transiently infects healthy individuals, particularly children, allowing this pathogen to persist. Thus, infectious *P. jirovecii* is widespread throughout the human population, and a large number of at-risk patients receive prophylaxis against PCP because they are very likely to be exposed [9,10,11,12,13]. PCP remains among the most common HIV-associated diseases [9,10,14,15], and recent trends indicate that the frequency of non-HIV-related PCP is rising significantly [16,17,18]. The adoption of certain immunomodulatory therapies for the clinical management of patients with inflammatory and autoimmune conditions and cancer has resulted in the appearance of PCP in patients previously not considered to be at risk [19,20,21]. In addition to its role as a primary cause of infectious pneumonia, *Pneumocystis* also exacerbates other forms of acute and chronic lung disease, including COPD and asthma [22,23,24,25,26]. *P. jirovecii* is included among the several fungal priority pathogens specified by the World Health Organization [27].

In order to cause disease, human fungal pathogens must either evade the human immune system, target immunocompromised patients, or both. Studies in human patients and animal models have demonstrated that intact adaptive immunity is required for effective host defense against *Pneumocystis* [28,29,30,31] and that immune defects that impair CD4^+^ T cell function or number place hosts at risk for developing PCP. Pulmonary innate immunity is typically not sufficient for protection against *Pneumocystis*, and immunocompetent humans and animals become transiently infected with *Pneumocystis* because of its ability to evade innate immunity through as-yet unknown mechanisms [32,33,34,35,36]. However, genetic determinants importantly influence this response [37]. Our prior work found that mice of the FVB/NJ inbred strain display effective innate immunity against *Pneumocystis murina* (*P. murina*), the species that infects mice [33,38], raising the possibility that a naturally occurring mechanism of pulmonary innate immunity against PCP exists. The discovery of mouse strains that are resistant to PCP provides an opportunity to compare how *Pneumocystis* interacts with the pulmonary innate immune system in susceptible and resistant mouse strains, with the goal of elucidating the specific mechanisms that lead to innate immune resistance. These important model systems may inform the design of novel antifungal treatments that either directly damage *Pneumocystis* or stimulate the innate immune system to enhance fungal recognition and eradication.

Mouse models of *Pneumocystis* infection have proven extraordinarily versatile and useful for the study of PCP in humans because of their close recapitulation of most aspects of human disease. Mouse models can be manipulated to reflect several underlying immunocompromised conditions that are known to render human patients susceptible to PCP. Furthermore, the existence of many distinct inbred laboratory mouse strains provides a platform to evaluate how host genetic diversity affects the life cycle and pathogenicity of *Pneumocystis* *in vivo*. In order to further investigate the rarity of effective antifungal innate immunity in genetically diverse laboratory mice, susceptibility testing was performed in additional inbred strains that are not closely related to the FVB/NJ strain. This work identified the AKR/J strain as exhibiting protective innate immunity against *Pneumocystis* infection even in the absence of T cell-mediated adaptive immunity. Additional studies revealed that functional alveolar macrophages are required for AKR/J resistance and that the resistance phenotype is passed to offspring in a dominant manner. These findings identify another mouse model to study how the initial host–pathogen interaction dictates the outcome of *Pneumocystis* infection and may provide insight into how *Pneumocystis* evades innate immunity in susceptible hosts.

## 2. Materials and Methods

### 2.1. Mice

All experimental mice were housed in the specific-pathogen-free colony at the University of Rochester. Animals received sterilized food and water, were housed in ventilated racks, and were handled using microisolator technology. Severe combined immunodeficient (SCID) mice on the C.B-17 background were co-housed with *Pneumocystis murina*-infected SCID mice and used as a source of infectious organisms for inoculations of experimental mice. AKR/J (JAX strain# 000648), BALB/cJ (JAX strain# 000651), C57BL/6J (JAX strain# 000664), DBA/1J (JAX strain# 000670), DBA/2J (JAX strain# 000671), 129S1/SvImJ (JAX strain# 002448), and FVB/NJ (JAX strain# 001800) were purchased from The Jackson Laboratory. Male and female animals were included. All research animals were treated in accordance with the regulations of the Association for Assessment and Accreditation of Laboratory Care International (AAALAC International). The research studies were approved by the University Committee on Animal Resources of the University of Rochester.

### 2.2. P. murina Infection

The lungs of heavily *P. murina*-infected SCID mice were perfused in situ through the right ventricle of the heart with PBS and surgically removed. Infectious *P. murina* organisms were then isolated from the dissociated lung tissue as previously described [39]. The final preparation was diluted, dropped on slides, and stained with Modified Grocott Methenamine Silver (Sigma-Aldrich, St. Louis, MO, USA) to enumerate cysts. In addition, *P. murina* preparations were cultured on chocolate blood agar plates to ensure the absence of contaminating microorganisms. Experimental mice were continuously depleted of CD4^+^ T cells by twice weekly intra-peritoneal injections of 0.3 mg/mouse of anti-mouse CD4 monoclonal antibody (clone GK1.5/TIB207, BioXCell, Lebanon, NH, USA) beginning 1 week prior to infection. Each mouse received 1 × 10^6^ *P. murina* organisms by direct intra-tracheal inoculation under isofluorane anesthesia. Some experimental mice were additionally depleted of CD8^+^ T cells by twice weekly intra-peritoneal injections of 0.25 mg/mouse of anti-mouse CD8 monoclonal antibody (clone 2.43/TIB210, BioXCell). The individual mice chosen to receive either control CD4-depletion or double CD4/CD8-depletion were selected randomly prior to any experimental manipulation. Each mouse then received 1 × 10^6^ *P. murina* organisms by direct intra-tracheal inoculation under isofluorane anesthesia. Mice were evaluated and weighed weekly following infection to determine whether any mice exhibited unexpected morbidity.

### 2.3. Alveolar Macrophage Depletion

Continuous depletion of resident alveolar macrophages was accomplished by once weekly intra-tracheal administration of clodronate-liposomes as we have described previously [33]. This technique has been used successfully to study alveolar macrophage function *in vivo* during *Pneumocystis* pneumonia and in other disease conditions [40,41,42]. Clodronate-liposomes or PBS-liposomes were synthesized at the University of Rochester according to published methods [43,44]. Control PBS-liposomes were prepared in an identical manner except that clodronate was omitted from the formulation. The liposomes were processed using sterile technique to minimalize the risk of contamination and were used within one week. The dosage required to deplete approximately 80% of the tissue resident AMs with a single dose was determined empirically. The clodronate-liposomes and PBS-liposomes were administered to experimental mice by direct intra-tracheal inoculation under isofluorane anesthesia. The individual mice chosen to receive either control PBS-liposomes or AM-depleting clodronate-liposomes were selected randomly prior to any experimental manipulation.

### 2.4. Determination of P. murina Lung Burden

The total lung *P. murina* burden of experimental mice was quantified by a real-time quantitative PCR (qPCR) assay as described previously [45]. A primer/probe set recognizing a portion of the single-copy *Pneumocystis murina* kexin gene was used in conjunction with a Bio-Rad CFX96 real-time PCR detection system (Hercules, CA, USA) for determination of total lung burden. The primer/probe sequences used were: forward primer, 5′-GCACGCATTTATACTACGGATGTT-3′; reverse primer, 5′-GAGCTATAACGCCTGCTGCAA-3′; and fluorogenic probe, 5′-CAGCACTGTACATTCTGGATCTTCTGCT TCC-3′. In addition, lung homogenates (LH) were stained with Modified Grocott Methenamine Silver Stain Kit (Sigma-Aldrich) to determine a direct visual quantification of *P. murina* asci and confirm the qPCR results. Briefly, triplicate 10 μL drops of each lung homogenate were air-dried and heat fixed. Following GMS staining according to the manufacturer’s instructions, cysts were counted in ten to fifty 100× oil immersion microscope fields (OIF). The total number of cysts were calculated by the following formula: (cysts/OIF) × (54,000 OIF/mL) × (Dilution Factor) × (Total Volume LH (mL)). GMS-stained slides were assigned a random identifier and counted in a blinded manner.

### 2.5. Flow Cytometry

For confirmation of antibody-mediated CD4-depletion in AKR/J mice, spleens were dissociated by forcing through a stainless-steel mesh in 10 mL of ice-cold RPMI 1640 media. The cells were passed sequentially through 70 micron and 40 micron filters, and red blood cells (RBC) were lysed with RBC lysis buffer (ThermoFisher, Waltham, MA, USA). Splenocytes were collected by centrifugation at 250× *g* for 10 min at 4 °C and washed with RPMI 1640 media. Recovered cells were resuspended in FACS buffer (1X PBS, 0.5% BSA, 0.05% sodium azide), incubated in Fc block (clone 2.4G2), and stained with anti-mouse CD4 mAb (clone RM4-4, BD Pharmingen, San Diego, CA, USA) and anti-mouse CD8 mAb (clone 53-6.7, BD Pharmingen) monoclonal antibodies. Importantly, the anti-mouse CD4 mAb used for flow cytometry (clone RM4-4) binds to a different epitope on mouse CD4 than the mAb used for depletion (clone GK1.5/TIB-207). A FACS LSRII machine (Becton Dickinson, Franklin Lakes, NJ, USA, University of Rochester Medical Center Flow Cytometry Core facility) was used to identify T cell subsets in isolated splenocyte populations. and data was analyzed with FlowJo v10.

### 2.6. Statistical Analyses

The significance of observed differences between experimental groups was determined using analysis of variance with Bonferroni’s multiple-comparison posttest (*p*-values of ≤0.05). Unpaired *t* tests were used for direct comparison of two groups. GraphPad Prism version 10.6.1 (GraphPad Software, San Diego, CA, USA) was used for statistical analyses. The numerical value used for the *P. murina* burden of animals without detectable organisms by qPCR or GMS staining was equivalent to the limit of detection of the specific assay. Based on our experience with the mouse model of *P. murina* infection, a sample size of 6–8 mice is sufficient to determine resistance or susceptibility to infection. Animals were not excluded from analyses unless they unexpectedly died prior to the established endpoint of the experiment and were not available for determination of *Pneumocystis* burden. The current studies involved continued depletion of CD4^+^ T cells, CD8^+^ T cells, or AM beginning prior to *P. murina* infection. Because these treatments given during the 5-week study timeline were to maintain the already depleted state, confounders related to treatment order were not intentionally controlled.

## 3. Results

### 3.1. CD4^+^ T Cell-Depleted AKR/J Mice Are Highly Resistant to Pneumocystis Infection

**Figure 1:**
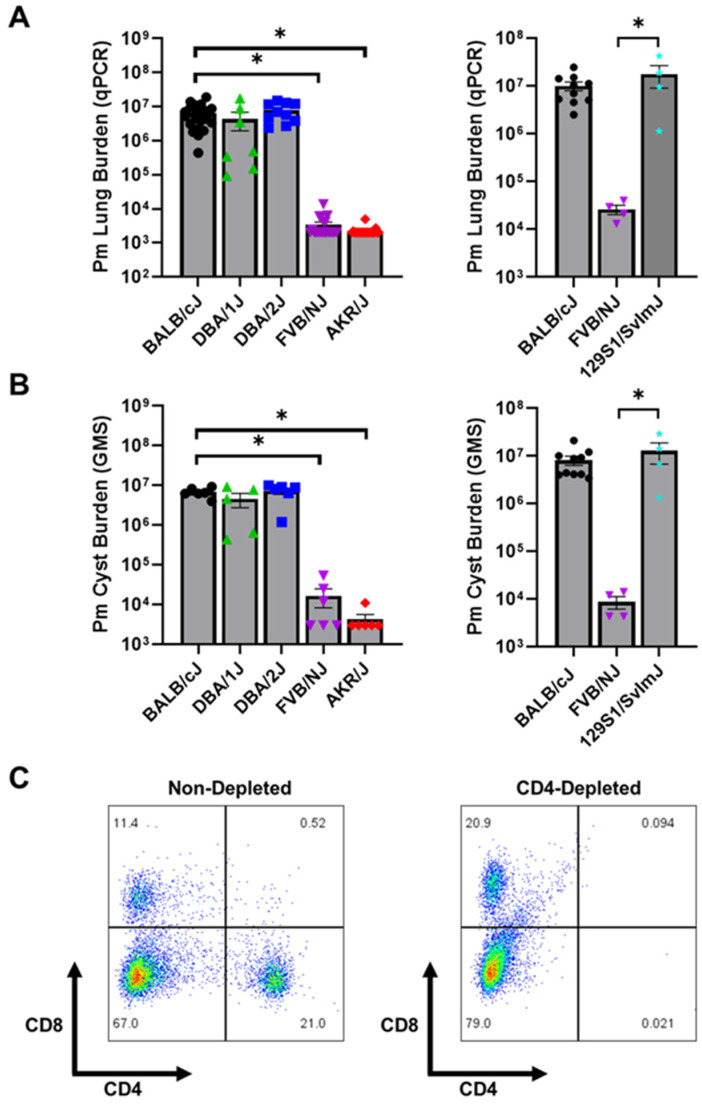
AKR/J mice display effective innate immunity against *P. murina* infection. CD4-depleted AKR/J (*n* = 22), BALB/cJ (*n* = 22), DBA/1J (*n* = 7), DBA/2J (*n* = 10), and FVB/NJ (*n* = 20) mice were intratracheally inoculated with 1 × 10^6^ freshly isolated *P. murina*. In a separate experiment, CD4-depleted BALB/cJ (*n* = 10), FVB/NJ (*n* = 4), and 129S1/SvImJ (*n* = 4), were also infected with *P. murina*. At 5 weeks post-infection the lungs of experimental mice were assessed for total *P. murina* burden by qPCR and asci counts by GMS stain (**A**,**B**). Mean ± 1 standard error measurement (SEM) are reported for each group. * denotes *p* < 0.05 compared to BALB/cJ mice. Flow cytometry was used to confirm the effective depletion CD4^+^ T cells in resistant AKR/J background mice (**C**).

Our prior work determined that, unlike most other inbred laboratory mouse strains, FVB/NJ mice remain resistant to *Pneumocystis murina* infection even in the absence of CD4^+^ T cell-dependent adaptive immunity [33,38]. In order to determine whether other genetically distinct mouse strains display effective innate immunity against *Pneumocystis*, several inbred strains not closely related to FVB/NJ were CD4-depleted and infected with *P. murina* via intra-tracheal inoculation. As expected, BALB/cJ mice were extremely susceptible to *P. murina* infection and harbored high fungal burdens as assessed by both real-time PCR quantification of *P. murina* DNA and direct counting of Grocott-Gomori Methenamine Silver (GMS)-stained cyst forms in lung homogenates (Figure 1A,B). CD4-depleted DBA/1J, DBA/2J, and 129S1/SVimJ were also found to be highly susceptible based on qPCR and GMS staining. In contrast, AKR/J mice displayed robust resistance to infection even in the absence of CD4^+^ T cells (Figure 1A,B). These mice had mean *P. murina* lung burdens that were near the limit of detection for the assay, and approximately three logs lower than the burden in susceptible BALB/cJ, DBA/1J, DBA/2J, and 129S1/SVimJ mice. Flow cytometry of the splenocyte population of CD4-depleted and non-depleted mice was used to confirm that anti-CD4 monoclonal antibody effectively depleted CD4^+^ T cells in resistant AKR/J background mice *in vivo* (Figure 1C). These data demonstrate that an additional mouse strain, AKR/J, displays innate resistance to *Pneumocystis* infection in the absence of functional adaptive immunity.

### 3.2. CD8^+^ T Cells Are Not Required for Effective Innate Immunity in AKR/J Mice

**Figure 2:**
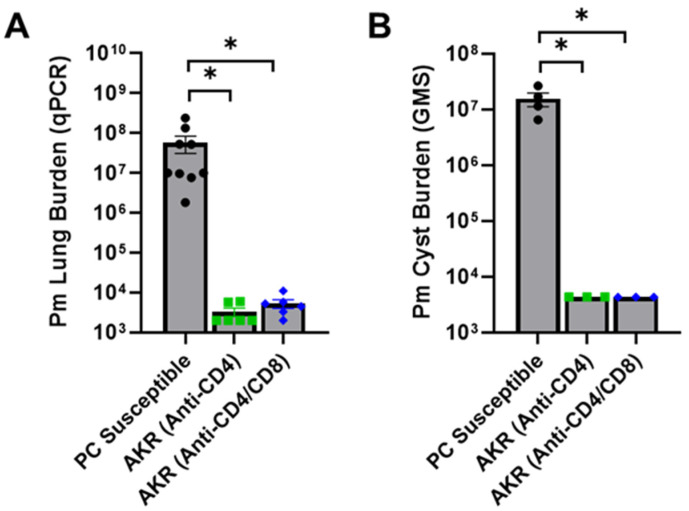
CD8^+^ T lymphocytes are not required for effective antifungal innate immunity in AKR/J mice. CD4-depleted (*n* = 6) and CD4/CD8 double-depleted (*n* = 6) AKR/J mice were intratracheally inoculated with 1 × 10^6^ freshly isolated *P. murina*. CD4-depleted BALB/cJ and C57BL/6J mice were also infected and used as *Pneumocystis*-susceptible controls (*n* = 9). At 5 weeks post-infection the lungs of experimental mice were assessed for total *P. murina* burden by qPCR and cyst counts by GMS stain (**A**,**B**). Mean ± 1 standard error measurement (SEM) are reported for each group. * denotes *p* < 0.05 compared to BALB/cJ mice.

Although CD8^+^ T cells do not typically provide meaningful host defense against *Pneumocystis* infection, previous reports have indicated that under certain circumstances CD8^+^ T cells can provide a degree of protection against *P. murina* in CD4-depleted mice [46,47]. Therefore, to determine whether AKR/J mice mount a distinct CD8^+^ T cell response that compensates for the loss of CD4^+^ T cell function and mediates protective immunity against PCP when CD4^+^ T cells are depleted, AKR/J mice were double depleted of both CD4^+^ and CD8^+^ T cells and then inoculated with *P. murina*. As expected, control *Pneumocystis* susceptible mice harbored large fungal burdens as assessed by qPCR and GMS staining (Figure 2). However, AKR/J mice remained robustly resistant to infection when either depleted of only CD4^+^ T cells, or double depleted of both CD4^+^ and CD8^+^ T cells (Figure 2). These data demonstrate that CD8^+^ T cells do not contribute to AKR/J resistance to respiratory fungal infection with *P. murina*, and suggest that T cell-independent innate immune mechanisms protect these mice from infection.

### 3.3. Alveolar Macrophages Are Required for AKR/J Resistance to P. murina Infection

**Figure 3:**
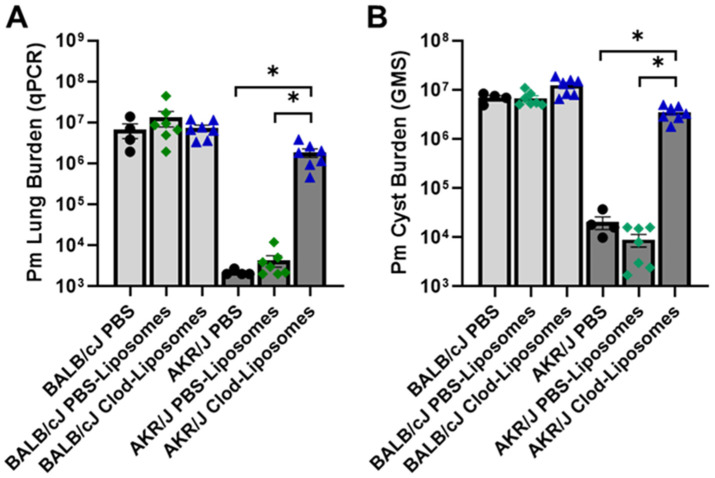
Alveolar macrophage (AM) depletion renders AKR/J mice susceptible to *P. murina* infection. CD4-depleted BALB/cJ and AKR/J mice were intra-tracheally instilled with either PBS-liposomes (*n* = 7) or AM-depleting clodronate-liposomes (*n* = 7) beginning one week prior to inoculation with 1 × 10^6^ freshly isolated *P. murina*. Control CD4-depleted BALB/cJ and AKR/J mice received PBS (*n* = 4). At 5 weeks post-infection the lungs of experimental mice were assessed for total *P. murina* burden by qPCR and cyst counts by GMS stain (**A**,**B**). Mean ± 1 standard error measurement (SEM) are reported for each group. * denotes *p* < 0.05 compared to BALB/cJ mice.

Lung-resident alveolar macrophages (AMs) are front-line innate immune cells that patrol the host–pathogen interface. While AMs contribute to pulmonary host defense against *Pneumocystis* [42,48,49], they are generally inadequate to protect against *Pneumocystis* pneumonia (PCP) in the absence of functional adaptive immunity [32]. In order to determine the contribution of the resident AM population to AKR/J innate immune resistance to *Pneumocystis*, mice were chronically depleted of AM using serial intra-tracheal administration of clodronate-liposomes, as we and others have described in previous studies [33,50,51]. Susceptible BALB/cJ and resistant AKR/J mice were subjected to double depletion of CD4^+^ T cell and AMs and then challenged with infectious *P. murina*. Control mice of each strain were CD4^+^ T cell-depleted and inoculated with either PBS or PBS-containing liposomes prior to challenge. The alveolar macrophage depletion efficiency achieved with clodronate-liposomes was approximately 80%. As expected, all susceptible BALB/c mice that were CD4^+^ T cell-depleted and treated with PBS, PBS-liposomes, or clodronate-liposomes developed high lung *P. murina* burdens after challenge, as confirmed by qPCR and cyst counts (Figure 3A,B). Also as expected, control CD4^+^ T cell-depleted AKR/J mice treated with PBS or PBS-liposomes remained resistant to infection. However, CD4^+^ T cell-depleted AKR/J mice that were also depleted of AMs with clodronate-liposomes became susceptible to *P. murina* infection (Figure 3A,B). These data demonstrate that alveolar macrophages are essential innate immune effectors for resistance against PCP in AKR/J mice. This contrasts with the typical ineffectiveness of AMs in providing protective host defense against PCP in hosts with diminished CD4^+^ T cell number or function.

### 3.4. The AKR/J Innate Resistance Phenotype Is Genetically Dominant

**Figure 4:**
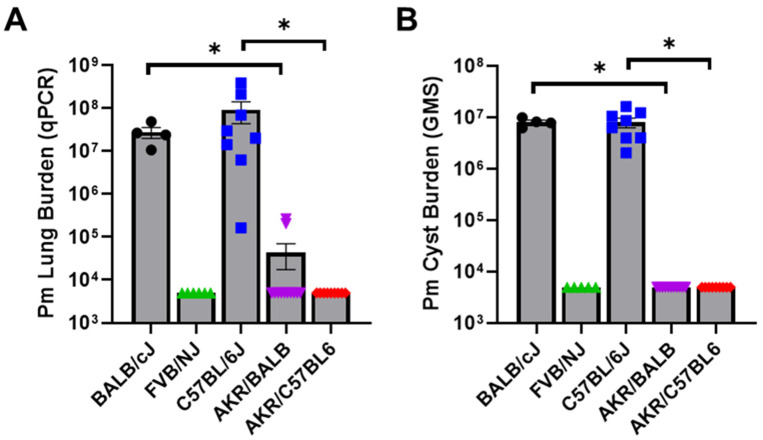
The AKR/J innate resistance phenotype is a genetically dominant trait. CD4-depleted BALB/cJ (*n* = 4), C57BL/6J (*n* = 8), FVB/NJ (*n* = 6), AKR/J x BALB/cJ F1 (*n* = 12) and AKR/J x C57BL/6J F1 (*n* = 9) mice were intratracheally inoculated with 1 × 10^6^ freshly isolated *P. murina*. At 5 weeks post-infection the lungs of experimental mice were assessed for total *P. murina* burden by qPCR and cyst counts by GMS stain (**A**,**B**). Mean ± 1 standard error measurement (SEM) are reported for each group. * denotes *p* < 0.05 compared to BALB/cJ mice.

In order to determine whether AKR/J innate resistance is a genetically dominant or recessive trait, F1 hybrids were generated by crossing susceptible BALB/cJ or C57BL/6J mice with resistant AKR/J mice. F1 hybrid mice were depleted of CD4^+^ T cells and challenged with *P. murina*. The BALB/cJ and C57BL/6J parent strains were used as controls. The fungal lung burden of experimental mice was determined by qPCR and GMS staining at 5 weeks post-infection. As expected, the BALB/cJ and C57BL/6J parent strains were both highly susceptible to *P. murina* infection when depleted of CD4^+^ T cells (Figure 4A,B). In contrast, both AKR/BALB and AKR/C57BL6 hybrid mice were highly resistant to *P. murina* infection (Figure 4A,B). These data suggest that a single copy of the critical genetic locus that regulates AKR/J antifungal innate immunity is sufficient for mice to express the resistant phenotype. Further genetic studies will be necessary to map this specific locus to a chromosomal location.

## 4. Discussion

*Pneumocystis* is widely recognized as a classic opportunistic fungal pathogen, primarily affecting individuals with compromised immune systems. In healthy humans and animals, *Pneumocystis* species are adept at evading early pulmonary innate immunity, allowing them to establish a limited local infection. However, this infection is typically transient, as the normal adaptive immune response effectively controls pathogen growth. This balance changes dramatically in individuals with diminished CD4^+^ T cell numbers or impaired CD4^+^ T cell function, such as patients with HIV/AIDS. In these immunocompromised hosts, *Pneumocystis* can cause severe and life-threatening *Pneumocystis* pneumonia (PCP), a condition that remains a significant cause of morbidity and mortality in this population. Despite its clinical importance, the precise mechanisms that enable *Pneumocystis* to evade the host’s innate immune defenses remain incompletely understood. This gap in knowledge represents a critical barrier to both fully understanding the pathogenicity of this fungal organism, and developing targeted therapeutic strategies. In this context, the current study highlights the discovery of an inbred mouse strain, AKR/J, which displays protective innate immunity against *Pneumocystis* infection, even in the absence of a functional adaptive immune system. This finding is particularly significant, as AKR/J is only the second mouse strain identified as resistant to *Pneumocystis* infection, along with the previously identified FVB/NJ strain. Importantly, AKR/J and FVB/NJ are phylogenetically distant inbred strains, with substantial genetic differences [52]. This genetic divergence raises the intriguing possibility that each strain employs a distinct mechanism to mount an effective innate immune response against *Pneumocystis*. Understanding these unique responses could provide valuable insights into the interplay between host genetics and immune defense mechanisms. Future research comparing the innate immune responses of resistant strains, such as AKR/J and FVB/NJ, to those of susceptible strains offers a promising avenue for uncovering the underlying mechanisms of effective innate immunity. Such studies could shed light on the specific factors that enable resistant strains to control *Pneumocystis* infection, as well as the vulnerabilities that lead to progressive infection in susceptible hosts. These findings could have broader implications for understanding host–pathogen interactions and may inform the development of novel therapeutic approaches to enhance innate immunity in immunocompromised individuals. By addressing these critical questions, researchers can take significant steps toward closing the knowledge gap surrounding *Pneumocystis* pathogenicity and improving outcomes for at-risk populations.

The current studies highlight alveolar macrophages as key players in the innate immune resistance to *Pneumocystis* observed in AKR/J mice. Although the administration of clodronate-liposomes only depleted approximately 80% of the resident alveolar macrophages, this level was sufficient to remove the natural innate immune protection of AKR/J mice and render them susceptible to PCP (Figure 3). While 20% of the AM population remained, they were unable to effectively control the infection. The most straightforward explanation for this finding would be that the smaller number of AM following clodronate treatment was not sufficient to patrol the large surface area of the lung, leaving unprotected niches where *Pneumocystis* could establish infection undetected. Alternatively, it is possible that AMs communicate with other cell types to initiate the innate immune cascade that eradicates *Pneumocystis*, and that the smaller number of AM remaining after clodronate-liposome treatments cannot generate a strong enough signal to produce an effective immune cascade. Further studies will be needed to determine the threshold number of AM required to maintain innate immunity in AKR/J mice.

The current findings demonstrating that alveolar macrophages are required for the effective innate resistance of AKR/J mice do not exclude the possibility that other innate immune mechanisms collaborate with alveolar macrophages to produce the resistant phenotype. For example, mice possess detectable levels of natural IgM antibodies that recognize multiple fungal pathogens, providing protection even in the absence of adaptive immunity [53]. Elevated levels of natural antifungal antibodies in AKR/J mice could potentially boost opsonization of *P. murina*, facilitating more efficient recognition and phagocytosis by resident alveolar macrophages. Alternatively, enhanced activity or differential polarization of pulmonary innate lymphoid cells (ILCs) in AKR/J mice might compensate for the absence of CD4^+^ T cells by releasing cytokines that activate resident alveolar macrophages for improved antifungal activity [54]. Alveolar epithelial cells (AT1 and AT2) are also known to regulate pulmonary innate immunity, and the life cycle of *Pneumocystis* requires attachment to the apical alveolar surface [55,56,57]. Therefore, crosstalk between alveolar epithelial cells and macrophages may further contribute to the innate resistance observed in AKR/J mice. While this study provides a foundation for future research comparing resistant and susceptible mouse strains, it also raises many additional important questions about the interactions and mechanisms that govern host susceptibility and resistance to *Pneumocystis*. 

Although *Pneumocystis* is classified as a fungal pathogen, traditional antifungal treatments are often ineffective against this opportunist. Instead, Bactrim (trimethoprim-sulfamethoxazole) is widely regarded as the gold-standard therapy for *Pneumocystis* pneumonia (PCP) and is also commonly used as a prophylactic measure for individuals at high risk of infection due to underlying conditions. Despite its efficacy, Bactrim presents certain limitations, and treatment failures and toxicity have been reported [58,59,60,61]. Furthermore, mutations that confer Bactrim resistance in other species have been identified in *P. jirovecii*, and the heavy reliance on a single therapeutic agent raises concerns about the potential emergence of antibiotic resistance [59,62,63]. These challenges underscore the urgent need for innovative strategies to address both treatment and prevention of PCP, as well as other fungal infections in humans. In our research, we have identified two genetically distinct inbred mouse strains that exhibit protective innate immunity against *Pneumocystis* infection. This discovery presents a unique opportunity to investigate the underlying mechanisms of resistance, with the goal of determining whether these processes can be leveraged for the development of novel *in vivo* treatments. Such studies have the potential to uncover innovative approaches, including strategies to directly damage *Pneumocystis*, inhibit fungal attachment within the lung, enhance macrophage-mediated recognition and phagocytosis of the pathogen, or disrupt its ability to acquire essential nutrients as an obligate biotroph. Advancing our understanding of these mechanisms could pave the way for transformative therapies that address the limitations of current antifungal treatments.

The discovery of protective innate immunity in certain mouse strains offers a promising avenue for understanding the complex host–pathogen interactions that govern *Pneumocystis* recognition and eradication. In resistant strains, innate immunity appears to play a pivotal role in controlling infection, whereas in susceptible strains immune evasion by *Pneumocystis* leads to progressive infection. This dichotomy highlights the pathogen’s ability to adapt to and evade host defenses. Notably, the requirement for adaptive immunity in most humans and mammals to mount an effective defense against *Pneumocystis* pneumonia suggests that the pathogen typically survives the initial encounter with innate immunity. Evidence supporting *Pneumocystis* immune evasion strategies includes findings that healthy, immunocompetent humans and rodents can experience transient infections [34,35]. These observations imply that *Pneumocystis* has evolved sophisticated mechanisms to bypass or suppress innate and adaptive immune responses, allowing it to persist in the host. Evasion of adaptive immunity by *Pneumocystis* species has been attributed to antigenic variation in the major surface glycoprotein (MSG)/glycoprotein A (gpA) family of surface proteins [64,65]. *Pneumocystis* possesses many distinct copies of the gene encoding MSG/gpA, and switching of the expressed isoform can alter the recognition of *Pneumocystis* organisms by antigen-specific T cells and antibodies. This antigenic modification of the *Pneumocystis* surface may help the pathogen avoid adaptive immunity long enough to initiate infection and ensure spread. However, there is little evidence that antigenic variation in MSG/gpA provides an advantage for *Pneumocystis* during its interaction with the innate immune system and alveolar macrophages. While the exact strategies of innate immune evasion are not completely understood, they may include direct suppression of innate immune cell activity or the ability to avoid detection by innate immune sensors. Previous studies have shown that the trophic form of *Pneumocystis* suppresses pro-inflammatory responses in innate immune cells [66,67]. Additionally, the MSG/gpA family of surface proteins plays a critical role in masking *Pneumocystis* β-glucan from detection, thereby impairing the activation of the innate immune system [68,69]. MSG/gpA proteins also directly suppress macrophage pro-inflammatory responses to β-glucan by interacting with inhibitory C-type lectins [70]. These combined mechanisms likely contribute to the ability of *Pneumocystis* to evade innate immunity in susceptible hosts and provide a foundation for exploring how macrophages from the resistant AKR/J mouse strain overcome innate immune suppression.

Unraveling the mechanisms by which *Pneumocystis* evades innate immunity could have significant implications for developing novel therapeutic approaches. Such insights could inform strategies to bolster innate immune defenses or disrupt the pathogen’s immune evasion capabilities, ultimately reducing the risk of infection in immunocompromised individuals. Advancing our understanding in this area is critical for improving outcomes in vulnerable populations and addressing the limitations of current prophylactic and therapeutic measures.

Although it is currently unclear whether AKR/J and FVB/NJ mice utilize similar or distinct mechanisms of innate antifungal resistance, there is compelling reason to speculate that these strains may have evolved diverse strategies to combat infection. Notably, AKR/J and FVB/NJ mice are each more closely related to several susceptible strains than they are to one another, making it unlikely that their resistance mechanisms stem from a shared ancestral trait. This genetic divergence suggests that their antifungal resistance may have arisen independently, potentially through distinct evolutionary pressures. A key difference between these strains lies in their immune profiles. AKR/J mice are generally associated with a type 1-biased immune response [71,72], while FVB/NJ mice exhibit a type 2-biased immune profile [33,73,74]. This distinction is supported by their responses to certain other pathogens. For instance, FVB/NJ mice demonstrate strong resistance to helminth infections, such as *Nippostrongylus brasiliensis* and *Trichuris muris*, which is consistent with their type 2 immune bias [75,76]. In contrast, AKR/J mice are permissive to these infections, likely due to their type 1 immune bias [71,77]. Furthermore, AKR/J mice, but not FVB/NJ mice, exhibit protective innate immunity against mousepox infection [78]. This resistance in AKR/J mice is associated with early IFNγ responses and natural killer cell activation and is genetically dominant [78,79,80]. Interestingly, our prior research demonstrated that IFNγ, while critical for AKR/J resistance to certain pathogens, suppresses host defense against *Pneumocystis* and renders resistant FVB/NJ mice susceptible to infection [33]. This finding underscores the complexity of immune responses and suggests that the mechanisms underlying resistance in AKR/J and FVB/NJ mice may be fundamentally different. The contrasting immune profiles and pathogen-specific responses of these strains highlight the potential for diverse pathways of innate antifungal resistance.

The identification of AKR/J as an additional resistant strain provides a valuable opportunity to further investigate the interaction between *Pneumocystis* and the pulmonary innate immune system. By comparing the resistance mechanisms of these two strains, future studies may uncover novel insights into the pathways that mediate innate immunity and identify potential targets for therapeutic intervention in immunocompromised hosts.

## Data Availability

The original contributions presented in this study are included in the article. Further inquiries can be directed to the corresponding author.

## Abbreviations

PCP	*Pneumocystis* Pneumonia.
PJP	*Pneumocystis jirovecii* Pneumonia
AM	Alveolar Macrophage
qPCR	Real-time quantitative polymerase chain reaction
HIV	Human Immunodeficiency Virus
Clodronate	Dichloromethylene diphosphonate
IFNγ	Interferon gamma
